# Clinical observation and electroretinogram analysis of ocular siderosis following ocular trauma: a retrospective case series

**DOI:** 10.3389/fmed.2025.1665613

**Published:** 2025-10-09

**Authors:** Yuyao Qu, Liwen He, Jixian Ma, Yaguang Hu, Jingming Li, Xiaolong Bai

**Affiliations:** ^1^Department of Ophthalmology, The First Affiliated Hospital of Xi’an Jiaotong University, Xi’an, Shaanxi, China; ^2^Department of Ophthalmology, Zhongshan Ophthalmic Center, Sun Yat-sen University, Guangzhou, Guangdong, China; ^3^Department of Ophthalmology, Yaozhou Zone People’s Hospital, Tongchuan, Shaanxi, China

**Keywords:** ocular siderosis, photopic electroretinogram (PH ERG), scotopic electroretinogram (SC ERG), ocular trauma, intraocular foreign body (IOFB)

## Abstract

**Background:**

To observe demographics and clinical characteristics of patients with ocular siderosis and to further analyze corresponding changes in electroretinogram (ERG) findings.

**Methods:**

The demographics and clinical characteristics from patients with ocular siderosis were collected and summarized, including sex, age, disease duration, visual acuity, and intraocular pressure. ERG parameters, including a-wave and b-wave latencies in photopic ERG and both amplitude and latency of b-wave in scotopic ERG, were collected. Paired comparisons were conducted to verify the difference between the affected eye and unaffected eye within the same individual.

**Results:**

A total of 15 patients with unilateral ocular siderosis were included, of whom 13 were male. The average age was 38.6 years. More than half of the affected eyes exhibited severely impaired visual acuity (≤0.02). Significant differences were observed between affected and unaffected eyes in the b-wave amplitude and latency in photopic ERG, b-wave amplitude in scotopic 0.01 ERG, and both a-wave and b-wave amplitudes and latencies in scotopic 3.0 ERG.

**Conclusion:**

Ocular siderosis is associated with marked retinal dysfunction, particularly involving the inner retinal layers. ERG proves to be a valuable tool for detecting and evaluating early retinal impairment in ocular siderosis, offering clinicians critical insights for timely diagnosis, management, and disease monitoring.

## 1 Introduction

Intraocular foreign body (IOFB), a severe complication of ocular trauma, can be caused by different types of accidents and injuries. Among IOFBs, metallic foreign bodies, particularly iron-containing IOFBs, may lead to a toxic reaction by releasing and repositioning iron ions in ocular tissues, which is termed ocular siderosis and first reported by Bunge in 1890, further analyzed by J F Ballantyne in 1954 (1–5). Studies have shown various ocular damages of ocular siderosis potentially leading to low vision, including retinal degeneration, cataracts, and glaucoma (6–8).

Currently, ocular as well as systemic examinations are the available methods for detecting ocular siderosis. Ocular examinations mainly include slit lamp examination, fundoscopy represented by scanning laser ophthalmoscopy (SLO), optical coherence tomography (OCT), and B-ultrasound. Systemic examinations, including computed tomography (CT) (9), provide existing evidence of metallic IFOBs from different perspectives, supporting the diagnosis of ocular siderosis. In addition to structural changes, functional tests are required to evaluate the retinal function and visual conduction pathway of the affected eye, such as visual evoked potential (VEP) and electroretinogram (ERG) (10–12).

ERG is a crucial tool for assessing retinal function, including photopic ERG (PH ERG) for cone cell function and scotopic ERG (SC ERG) for rod cell functional detection (13, 14). According to the International Society for Clinical Electrophysiology of Vision (ISCEV) standards, ERG includes full-field electroretinogram (ff-ERG), pattern electroretinogram (pattern ERG or PERG), multifocal electroretinogram (multifocal ERG or mf-ERG) (15). A typical ERG response consists of a small negative wave, known as a-wave, followed by a larger positive wave, known as b-wave, corresponding to the hyperpolarization of the photoreceptors and the depolarization of bipolar cells, respectively. These ERG parameters are quantified by amplitude and latencies.

Application of ERG for appraising ocular siderosis has gained attention for years, particularly in assessing retinal functional damage and predicting visual prognosis (16). Existing reviews report that reduction in b-wave amplitude may occur in the early stages of ocular siderosis (9). Previous case report of Angeline L Wang showed a reduction of the b-wave amplitude (17). Zhigang LV reported the diminished ERGs in both a-wave and b-wave of a patient with ocular siderosis; the same result was demonstrated in Adriana Berezovsky as well (18, 19). Moreover, Sahay *et al*. found mf-ERG may reveal subclinical electrophysiological retinal dysfunction in eyes with iron IOFB (20). However, analysis of ff-ERG about ocular siderosis has some defects. More attention should be drawn to evaluate the damage of retinal function and visual conduction pathway via ERG analysis, especially a-wave and b-wave changes in ff-ERG.

In this study, we analyzed ERG results in patients with ocular siderosis, revealing the characteristics of retinal functional changes and discussing their clinical significance in diagnosis and treatment, providing a theoretical basis for improving prognosis.

## 2 Methods

This is a retrospective case series of ocular siderosis observed in Zhongshan Ophthalmic Center, Sun Yat-sen University. This study was conducted in accordance with the Declaration of Helsinki, and ethical approval was obtained from the Institutional Review Board of the hospital. Inclusion criteria were eyes with ocular siderosis induced by metallic foreign bodies, patients who underwent ff-ERG before receiving any surgical treatments, and ERG recordings of acceptable technical quality. Exclusion criteria were affected eyes previously diagnosed with other ocular diseases, such as cataract, glaucoma, vitreoretinopathy; patients with unsymmetrically best corrected visual acuity of both eyes ahead of injury; and patients who did not undergo ERG examination. The cases were identified from electronic medical records and case files retrieved. Demographic data, duration of time from ocular injury to diagnosis of ocular siderosis, clinical findings were subsequently extracted and analyzed from the case files.

All patients had detailed ocular examination including visual acuity (VA) assessment, noncontact intraocular pressure, slit lamp examination of the anterior segment, dilated indirect fundus examination, imaging examination, and ERG.

The diagnosis of ocular siderosis in this study was established based on the following criteria: (1) a documented history of IOFB exposure or trauma; (2) clinical manifestations, including progressive visual decline, lens changes (such as cataract), and fundus alterations; (3) and supporting examinations such as imaging evidence of residual IOFB (such as SLO, B-ultrasound and CT). All enrolled cases met the above diagnostic criteria, ensuring comparability within the study cohort.

### 2.1 Examination procedures of ERG examination

Scotopic and photopic ERG were performed separately on both eyes (Roland consult, Germany). The recording was done using skin electrodes. A positive electrode was placed over the lower eyelid, while a negative electrode at the outer canthus. A ground electrode was placed at the ipsilateral earlobe. Skin was cleaned, and conductive paste (integral to the electrode) was applied to ensure stable electrical connections. All the ERG examining procedures followed the protocol of the International Society for Clinical Electrophysiology of Vision (ISCEV) Standard for ff-ERG (2022 update) (21). The results of ERGs were evaluated by two different professional ophthalmic technicians.

### 2.2 Statistical analysis

Statistical analyses were performed using IBM SPSS Statistics for Windows, version 27.0 (IBM Corp., Armonk, NY, USA) and GraphPad Prism 9.5 (GraphPad Software, La Jolla, CA). The distribution of continuous numerical data was assessed by the Shapiro–Wilk normality test. For continuous variables with normal distribution, mean and standard deviation (SD) were calculated. For continuous variables that did not follow normal distribution, medians, 25% and 75% percentiles were calculated. Wilcoxon test or paired *t*-test were used to compare ERG characteristics between the affected eye and the healthy eye. A *p-value* less than 0.05 was considered statistically significant.

## 3 Results

### 3.1 Demographics of patients with ocular siderosis

A total of 15 patients were included, all of whom had ocular siderosis in a single eye. Among them, three patients had cornea siderosis, while nearly half (7 of 15) eyes had lens siderosis. Demographics of patients are shown in detail in Table 1.

**Table 1:** Demographics of participants with single eye affected with ocular siderosis.

Characteristics	Median or mean*	Test of normality	25% percentiles	50% percentiles	75% percentiles
Gender (Male No., %)	13 (86.7%)				
Age (Years)	38.6	*p* = 0.505	26	37	48
Disease Duration (Months)	12	*p* < 0.001	6	12	24
Visual acuity (decimal)	0.02	*P* < 0.001	<0.02	0.02	0.3
IOP (mmHg)	10.7	*P* < 0.001	10.0	10.7	14.7

*: Means or medians were calculated for variables obey or disobey normal distribution.

**Figure 1:**
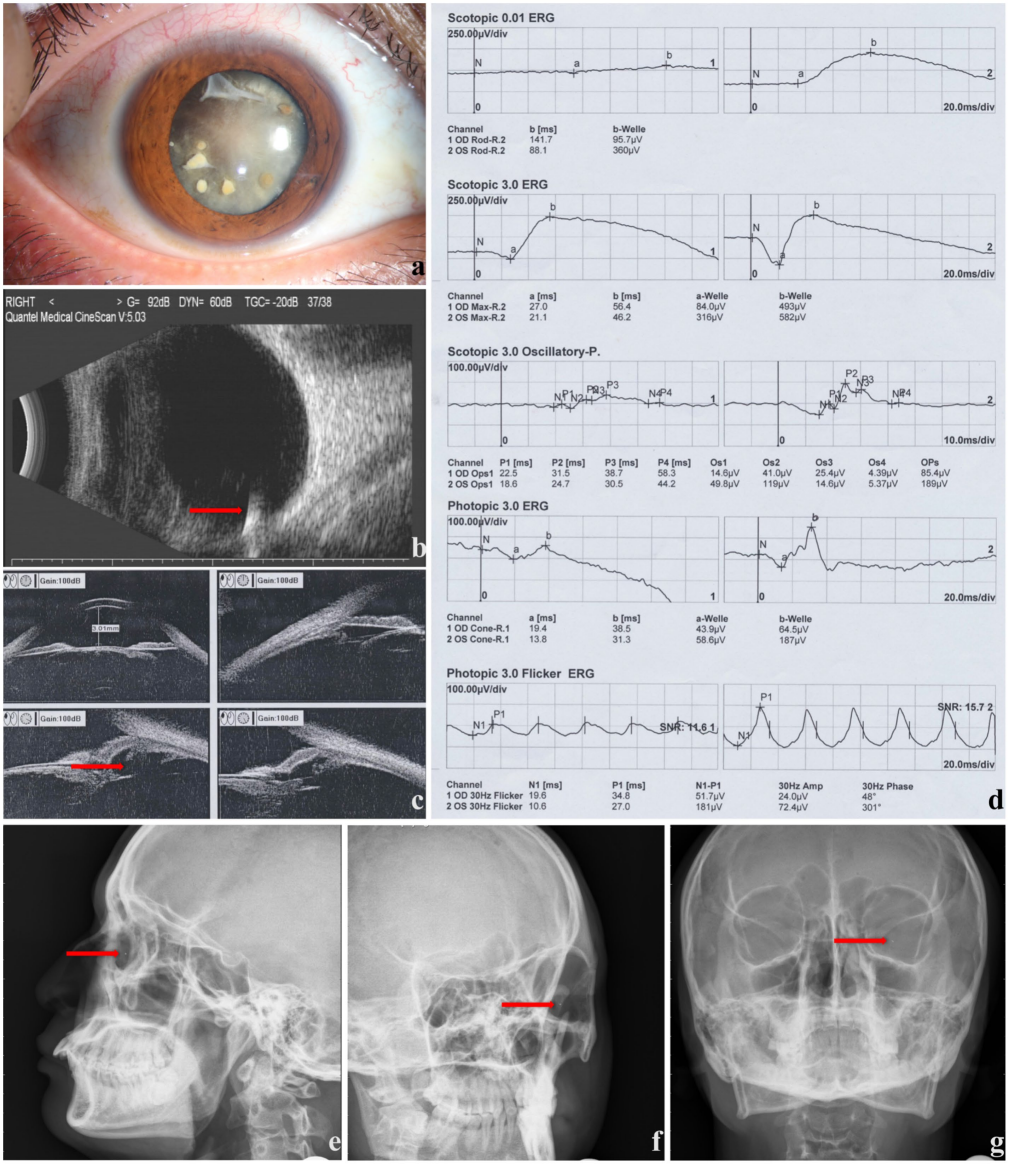
The ophthalmic examinations of a male patient with ocular siderosis (right eye involved). **(a)** Anterior segment photography presenting corneal and lenticular involvement. **(b)** B-ultrasound showing foreign body in vitreous body (red arrow). **(c)** Ultrasound biomicroscope showing ciliary body rupture (red arrow). **(d)** ERG results, **(e–g)** X-ray images showing foreign body (red arrow).

Representative ophthalmic examination results are shown in Figure 1.

### 3.2 ERG results of patients with ocular siderosis

A total of 26 well-qualified ERGs of 15 patients were collected, including 13 paired eligible PH ERGs and 13 paired SC ERGs.

In PH ERG results, amplitude of a-wave in control eyes, latency of b-wave in control eyes, amplitude of b-wave in affected eyes and control eyes were normally distributed (*p* = 0.829, *p* = 0.934, *p* = 0.194, and *p* = 0.559, respectively). In contrast, the latency of a-wave in affected and control eyes, the amplitude of a-wave in affected eyes, and the latency of b-wave in affected eyes did not meet the assumption of normality (*p* < 0.05).

In SC 0.01 ERG results, the latency of b-wave in affected eyes and control eyes did not meet normal distribution (p < 0.05), whereas the amplitude of b-wave in affected eyes and control eyes met normal distribution (*p* = 0.498 and *p* = 0.527, respectively). In SC 3.0 ERG, the latency of a-wave in control eyes, the amplitude of a-wave in affected eyes and control eyes, the latency of b-wave in control eyes, and the amplitude of b-wave in both affected eyes and control eyes did not meet normal distribution (*p* < 0.05).

The descriptive statistics results showed that means or medians of the latencies in affected eyes were longer than those in control group (Table 2). Meanwhile, means or medians of the amplitudes in affected eyes were lower than those in control group (Table 2) Notably, the value of b-wave amplitudes in affected eyes for both SC 0.01 ERG and SC 3.0 ERG were more than two times those of the control eyes. Detailed characteristics of parameters are shown in Table 2.

**Table 2:** Characteristics of parameters in ERG results.

Variables of ERG		Affected eye*	Control eye*	Wilcoxon test *Z* value	Paired tests**	r***
PH ERG	Latency of a-wave (mS)	18.0 (15.4, 22.0)	16.0 (15.0, 17.0)	−1.632	*p* = 0.103	−0.453
	Amplitude of a-wave (μV)	30.6 (16.3, 69.5)	46.0 ± 5.2	−1.293	*p* = 0.196	−0.359
	Latency of b-wave (mS)	36.0 (31.2, 37.9)	32.2 ± 0.6	−2.100	***p* = 0.036**	**−0.583**
	Amplitude of b-wave (μV)	70.4 ± 16.3	138.3 ± 12.5	−2.970	***p* = 0.003**	**−0.824**
SC 0.01 ERG	Latency of b-wave (mS)	87.0 (39.9, 99.6)	86.0 (82.2, 91.5)	−0.510	*p* = 0.610	−0.141
	Amplitude of b-wave (μV)	55.5 ± 27.3	220.2 ± 12.0	−3.180	***p* = 0.001**	**−0.882**
SC 3.0 ERG	Latency of a-wave (mS)	25.0 (23.3, 34.1)	22.1 ± 0.6	−2.764	***p* = 0.006**	**−0.767**
	Amplitude of a-wave (μV)	137.3 ± 26.7	223.5 ± 21.4	−2.830	***p* = 0.005**	**−0.785**
	Latency of b-wave (mS)	52.4 (46.0, 58.5)	51.2 ± 1.4	−0.510	*p* = 0.610	−0.141
	Amplitude of b-wave (μV)	178.1 ± 38.5	407.7 ± 255.2	−2.970	***p* = 0.003**	**−0.824**

*: For variables obey normal distribution and means ± standard deviations (SDs) were calculated. For variables disobey normal distribution (p < 0.05), medians and 95% were calculated. **: Paired *t-*test or Wilcoxon test was used for variables obey or disobey normal distribution. ***: Calculated as Z/√N, in this study, *N* = 13.

Furthermore, the paired test showed statistically significant differences in PH ERG, statistically significant differences were found in b-wave latency and amplitude. In SC 0.01 ERG, statistically significant differences were found in amplitude of b-wave. In SC 3.0 ERG, statistically significant differences were found in a-wave latency and amplitude, as well as in the amplitude of b-wave. Further detailed paired testing results are shown in Table 2.

By calculating r value, the effective sizes of b-wave in SC ERG were relatively strong. In particular, the amplitudes in SC 0.01 ERG and SC 3.0 ERG reached over 0.5 with absolute value −0.882 and −0.824, respectively. In addition, in PH ERG, the latency and amplitude of b-wave presented strong effective sizes, with r values of −0.583 and −0.824, respectively. As for a-wave, the strong differences are mainly showed in SC ERG. However, though there is no significant difference found in a-wave of PH ERG between affected and fellow eyes, r value indicates the trend of difference is relatively strong, which is worth paying more attention to. Further detailed statistics of r value are shown in Table 2.

## 4 Discussion

In this study, we retrospectively analyzed 15 patients with ocular siderosis. The demographic findings in our results suggest that ocular siderosis can have various clinical manifestations, resulting in significant damage on retinal function and visual conduction pathway. The vision loss in ocular siderosis is consistent with previous studies reporting various ocular damages, including cornea opacity and cataract, which can result in low vision (22–24).

Ocular siderosis is caused by the release and redistribution of iron ions from an intraocular metallic foreign body. The toxic chain reactions triggered by such foreign bodies should not be underestimated. Previous studies have demonstrated that excess iron is toxic to the retina, and numerous investigations have explored the mechanisms of iron-induced retinal damage. T Hiramitsu et al. reported that lipoperoxide formation in the retina in the presence of iron released from a piece of iron inserted into the vitreous, resulting in retinal degeneration (25). In addition, iron has been shown to induce inflammation in the retina, contributing to retinal toxicity (26). While previous research have confirmed that excessive iron can be toxic to photoreceptor cells and retinal pigment epithelium (RPE) cells *in vivo*, studies specifically focusing on photoreceptor cell changes as well as statistical data derived from human subjects are limited (27).

As mentioned above, various ocular damages, including retinal functional changes caused by ocular siderosis, can complicate diagnosis (28). Although SLO, OCT, B-ultrasound, and ocular CT can help confirm the existence of a foreign body, further investigation is needed to assess ocular dysfunction, particularly vitreoretinal damage, which may have determinative impact on visual acuity. ERG is a non-invasive tool for not only objectively assessing retinal function but also indirectly reflecting the alteration of photoreceptors, bipolar cells, and retinal ganglion cells (29). However, characteristics of ERG results in ocular siderosis still require further research. In this study, we used ERG variables as parameters to investigate the alteration (30).

In ocular siderosis, iron ions would continuously release and accumulate unless the metallic foreign body is taken away. Moreover, clinical changes may not match with the damage degree of retinal function. Thus, investigations such as ERG could provide a better understanding of disease progression (31). In the present study, regarding the ERG results, we found statistically significant differences between the affected eye and the healthy eye in ERG b-wave results, including amplitude and latency in PH ERG, amplitude in SC 0.01 ERG, and the amplitude in SC 3.0 ERG, which align with the conclusion in the review of Martina Menchini (9). There are various hypothesis and theories debating on the origination of b-wave in ERG (32). The commonly accepted theory in recent years is that b-wave originates from retinal bipolar cells and reflects the firing patterns of depolarizing rod bipolar cells (DBC_R_) (33–35). DBC_R_, which depolarizes in response to light spots, are found to have synaptic contact with rod photoreceptors in mammalian retinas (36, 37). In the current study, however, the result implies that patients with ocular siderosis experience severe degeneration of retinal inner neurons.

In addition, the statistical differences are shown significantly in a-wave. In this study, the main discussion focuses on the SC 3.0 ERG results, where both latency and amplitude of a-wave are showing differences among groups. Comparison of distributions between groups showed that in affected eyes, the latencies were longer, and the amplitudes tended to decline. Evidence shows that SC 3.0 ERG reflects mixed rod and cone system responses, and rod system contribution dominates in healthy retina. The results indicated the impairment of the retinal photoreceptor cells (21, 38). As shown in previous studies, retinal photoreceptors transform photon energy into electrical signals through a cascade of biochemical reactions, a process referred to as phototransduction. In dark-adapted photoreceptors, guanylate cyclase (GC) synthesizes cyclic guanosine monophosphate (cGMP) from guanosine monophosphate (GMP), while phosphodiesterase catalyzes the hydrolysis of cGMP into GMP. The dynamic equilibrium between the synthesis and degradation of cGMP maintains this balance (39, 40). Deepak K. Pattanaik demonstrated that the presence of iron ions generates reactive oxygen species (ROS), increases the calcium flux, and results in reduction in the amplitude and slope of the a-wave voltage in the electroretinogram (39, 41). Furthermore, caspase is involved in the reaction, as reported by Nuria Sanvicens in a study on oxidative stress-induced apoptosis in retinal photoreceptor cells (42).

Although no significant difference was found in the a-wave of PH ERG between affected and fellow eyes, calculation of R value revealed a relatively strong trend suggesting potential differences. These findings align with the study by P A Sieving, which focuses on the early receptor measurements of ocular siderosis (43). The presented insignificant differences may be attributable to insufficient sample size and worth paying more attention to in our future study.

Prognosis of ocular siderosis can be various. Combining multi-pattern radiology examinations and functional tests could relatively contribute to clinical diagnosis and treatment planning. There is a study focusing on ERG changes and post-surgery visual prognosis of patients, demonstrating the vital significance of ERG examination in ocular siderosis (44). Early removal of the intraocular foreign body is crucial to prevent or minimize the progression of ocular siderosis (45). However, our study has some limitations. The sample size is relatively small, which may limit the generalizability of the results. Future studies with larger cohorts are needed to validate these results. Another limitation of this study is the lack of systematic data regarding surgical management (such as intraocular foreign body removal and its timing) and long-term prognosis. Although we analyzed the ERG results comprehensively, other factors that may affect ERG results such as the duration of ocular siderosis and the treatment history of patients should be addressed in future prospective investigations.

This study systematically combined ERG findings with clinical assessment in patients with ocular siderosis associated with intraocular foreign bodies. Unlike previous reports that primarily focus on individual case descriptions or imaging features, our work quantitatively characterizes retinal dysfunction and highlights the potential of ERG as a sensitive diagnostic tool, even in cases with variable initial clinical presentations. By providing objective measures of retinal impairment, this study offers novel insights into early detection and assessment of ocular siderosis, which may aid clinicians in timely diagnosis and management.

## 5 Conclusion

Our study provided valuable insights into the characteristics of retinal functional changes in patients with ocular siderosis, as revealed through ERG analysis. We identified characteristic alterations in retinal function that may reflect underlying cellular damage, particularly involving bipolar cells. These findings not only highlight the potential role of bipolar cell apoptosis in the pathophysiology of ocular siderosis but also provide important data that could be leveraged for artificial intelligence (AI)-based diagnostic models, offering a probable direction to future research. By integrating ERG-derived functional patterns into AI algorithms, it may become feasible to achieve earlier and more accurate recognition of ocular siderosis, as well as automated risk stratification. Clinically, such approaches may support more accurate diagnosis and timely intervention, ultimately aiding in the preservation of visual function. To further enhance our understanding and improve patient outcomes, future studies are warranted to involve larger sample sizes and consider additional influencing factors that may affect ERG results, such as time course, which would enable the development and validation of robust AI systems trained to incorporate ERG results alongside other clinical parameters. This study underscores the dual value of functional retinal assessment; not only as a crucial tool in the comprehensive management of patients with intraocular metallic foreign bodies but also as a critical component for developing intelligent diagnostic and decision-support tools in ophthalmology.

## Data Availability

The original contributions presented in the study are included in the article/supplementary material. Further inquiries can be directed to the corresponding authors.

## Glossary

IOFB: intraocular foreign body
SLO: scanning laser ophthalmoscopy
OCT: optical coherence tomography
CT: computed tomography
VEP: visual evoked potential
ERG: electroretinogram
PH ERG: photopic electroretinogram
SC ERG: scotopic electroretinogram
ISCEV: International Society for Clinical Electrophysiology of Vision
ff-ERG: full-field electroretinogram
PERG: pattern electroretinogram
mf-ERG: multifocal electroretinogram
VA: visual acuity
RPE: retinal pigment epithelium
DBC_R_: depolarizing rod bipolar cells
GC: guanylate cyclase
cGMP: cyclic guanosine monophosphate
ROS: reactive oxygen species
AI: artificial intelligence
